# Safety and efficacy of endovascular thrombectomy in acute ischemic stroke treated with anticoagulants: a systematic review and meta-analysis

**DOI:** 10.1186/s12959-022-00394-y

**Published:** 2022-06-21

**Authors:** Jia-Hung Chen, Chien-Tai Hong, Chen-Chih Chung, Yi-Chun Kuan, Lung Chan

**Affiliations:** 1 Department of Neurology, Shuang-Ho Hospital, Taipei Medical University, New Taipei City, Taiwan; 2 Department of Neurology, School of Medicine, College of Medicine, Taipei Medical University, Taipei, Taiwan

**Keywords:** Endovascular thrombectomy, Anticoagulants, Symptomatic intracranial hemorrhage

## Abstract

**Background:**

Endovascular thrombectomy (EVT) is an effective therapy in acute ischemic stroke (AIS) with large vessel occlusion, especially for those who are unsuitable for intravenous thrombolysis. However, the safety and efficacy of EVT in AIS patients who receiving oral anticoagulants (OACs) is unclear, especially for the risk of symptomatic intracranial hemorrhage (sICH).

**Methods:**

Database of PubMed, Embase, and Cochrane Library were searched from Jan 1, 2000, through the final search date of Jun 2, 2021. Eligible studies for enrollment required outcomes reported for events of sICH, mortality, functional status, and successful reperfusion. Meta-analysis was conducted to compare the outcomes difference after EVT between AIS patients with or without OACs use. The primary safety outcome was sICH after EVT, and the primary efficacy outcome was functional status at 3 months.

**Results:**

One thousand nine hundred forty studies were screened for eligibility and 15 of them were included in the meta-analysis. Compared the OACs group to control arm, vitamin K antagonists (VKAs) was associated with higher risk of sICH (OR 1.49, 95% CI 1.10–2.02) and mortality (OR 1.67, 95% CI 1.35–2.06). Poor functional outcomes were noted both in the VKAs and direct oral anticoagulants (DOACs) groups (OR 0.62, 95% CI 0.54–0.71 and OR 0.61, 95% CI 0.53–0.71, respectively). No differences in successful reperfusion were observed.

**Conclusions:**

Comparing with DOACs, VKAs use was associated with a higher risk of sICH and mortality after EVT. Patients who did not receive OACs exhibited more favorable outcomes. The successful reperfusion did not differ between groups. However, results for mortality and functional outcomes have to be interpreted with caution since they are based on non-randomized data and unadjusted proportions.

**Supplementary Information:**

The online version contains supplementary material available at 10.1186/s12959-022-00394-y.

## Background

Oral anticoagulants (OACs) including vitamin K antagonists (VKAs) and direct oral anticoagulants (DOACs) are widely used today among people with atrial fibrillation, venous thrombosis, mechanical valve replacement, and autoimmune disorders such as antiphospholipid syndrome or vasculitis [1, 2]. However, people taking OACs who suffered from acute ischemic stroke (AIS) are usually disqualified from intravenous recombinant tissue plasminogen activator (rtPA) according to current guideline [3]. Endovascular thrombectomy (EVT), therefore, remain an effective therapy for AIS patients receiving OACs and present with large vessel occlusion (LVO).

Symptomatic intracranial hemorrhage (sICH) is a disaster complication of EVT. AIS patients who under OACs treatment and receiving EVT may taking higher risk of sICH since its stronger anticoagulative effects. In the HERMES (Highly Effective Reperfusion Evaluated in Multiple Endovascular Stroke) study, the incidence of sICH was reported to be 4.4% in pooled analysis from five large randomized control trials [4]. However, they did not differentiate the risks of sICH for patients receiving and not receiving OACs, nor specifically investigate the mortality and efficacy in these two groups. There are also lack of randomized data and studies are scare and mostly observational. In the present meta-analysis, we investigated the safety and efficacy of EVT in patients with AIS receiving anticoagulants or a confirmed therapeutic dose of OACs.

## Material and methods

This systematic review and meta-analysis in accordance with the Preferred Reporting Items for Systematic Reviews and Meta-Analyses guidelines. The review protocol was registered with PROSPERO (International Prospective Register of Systematic Reviews) (CRD42021273951).

### Eligibility criteria and study selection

This systematic review was performed by two reviewers (J.H.C. and Y.C.K.), and any disagreements were resolved after a panel discussion involving three reviewers (J.H.C., Y.C.K., and L.C.). After duplicate studies were removed, the titles and abstracts of the remaining papers were reviewed, and the full texts of potentially eligible studies were evaluated. The inclusion criteria were as follows: (1) involvement of patients with AIS who underwent EVT, regardless of whether it was combined with intravenous rtPA; (2) patients with pretreatment anticoagulant use (VKAs or DOACs); (3) randomized controlled or retrospective study design with at two or more comparator arms; and (4) data on sICH events, mortality, functional outcomes, or reperfusion rate. All duplicate studies, crossover trials, uncompleted clinical trials, review articles, and studies that did not use original data were excluded. No limitations were placed on the publication language.

### Information sources and search strategy

The PubMed, Embase, and Cochrane Library databases were searched from Jan 1, 2000, through the final search date of Jun 2, 2021. The literature search was conducted using keywords related to endovascular procedures, mechanical thrombectomy, anticoagulation, VKAs, DOACs, and intracranial hemorrhage. The reference sections of prior systematic reviews and meta-analyses were also screened for related studies. The search strategy is detailed in Supplementary Table 1.

### Data collection

Baseline characteristics, intervention data, medication profiles, and outcome data were independently extracted by two reviewers (J.H.C. and Y.C.K.). Information on study designs, study population, and inclusion and exclusion criteria was also retrieved. Any disagreements regarding the collected data were reconciled through discussion with a third reviewer (L.C.).

### Outcome measures

The primary safety outcome was sICH after EVT, which was determined according to the European Cooperative Acute Stroke Study II criteria, hemorrhagic infarction type 1 to parenchymal hematoma type 2 classification, National Institute of Neurological Disorders and Stroke criteria, or Safe Implementation of Thrombolysis in Stroke-Monitoring Study criteria. The secondary safety outcome was mortality at 3 months. The primary efficacy outcome was functional status according to the modified Rankin scale score at 3 months. The secondary efficacy outcome was the successful reperfusion rate according to the modified treatment in cerebral infarction (mTICI) score. If more than one definition of sICH was used in a single study, the most common definition was used for outcome analysis.

### Risk of bias assessment

Two independent reviewers (J.H.C. and Y.C.K.) assessed the risk of bias (low, intermediate, or high) of the included studies using the Risk of Bias in Non-randomized Studies of Interventions tool. Any disagreements were resolved after a panel discussion involving all three reviewers (J.H.C., Y.C.K., and L.C.). The results of the quality assessment are provided in the Supplemental Material.

### Statistical analysis

All analyses were conducted using Review Manager 5.4 (The Cochrane Collaboration, Oxford, UK). The summary effect sizes for outcomes in the VKA and DOAC groups versus patients (controls) who did not receive OACs were estimated using the DerSimonian and Laird random-effects model. Statistical significance was indicated 95% confidence intervals (CIs) that did not cross 1. The heterogeneity and inconsistency across studies were assessed using the I^2^ statistic. Publication bias was evaluated through visual inspection of funnel plots (provided in the Supplemental Material).

## Results

### Search results and study characteristics

**Fig. 1:**
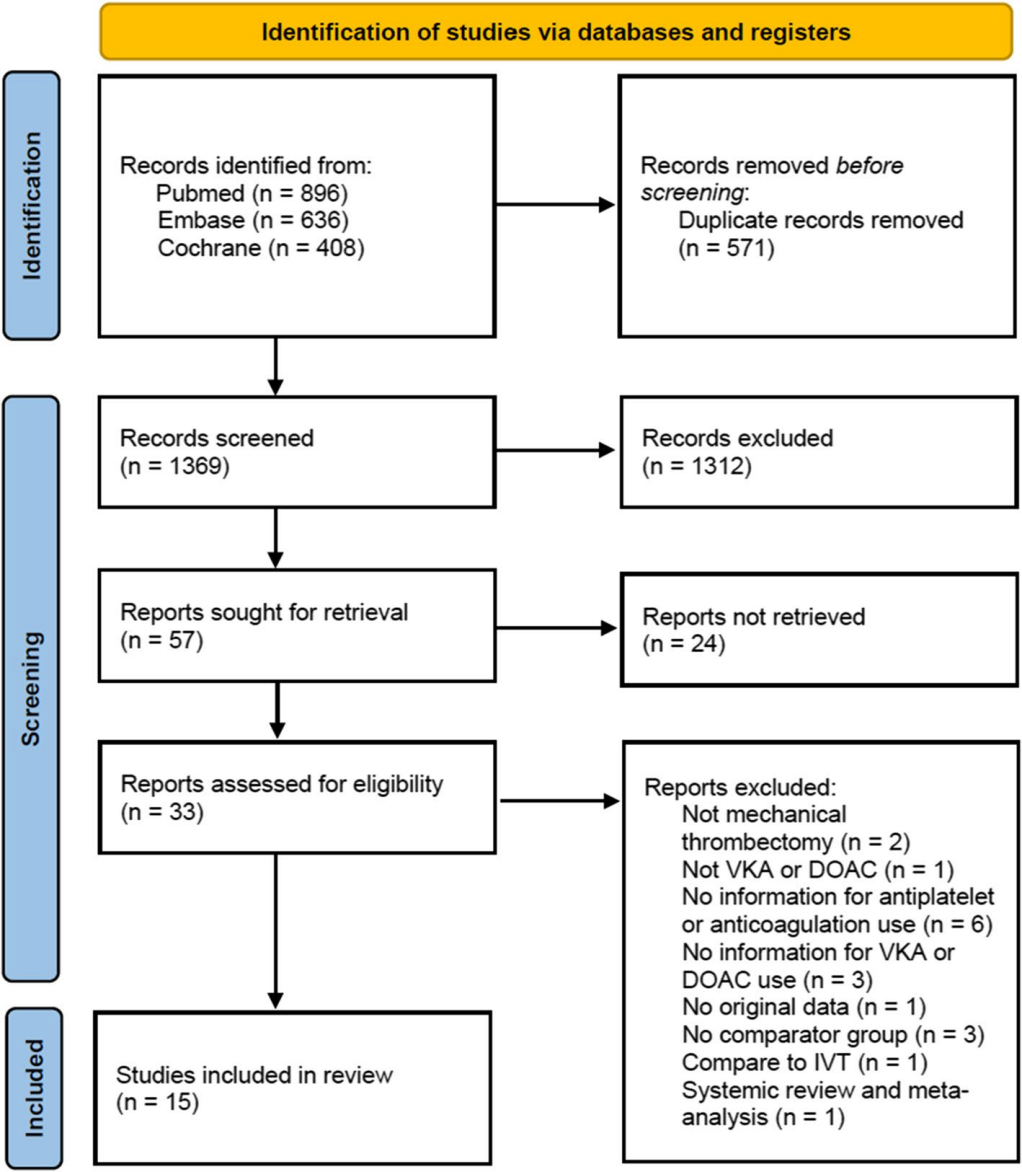
Study selection flow chart

The database search yielded 1940 studies. After 571 duplicate studies were removed, title and abstract screening led to the exclusion of an additional 1312 studies. The remaining 57 studies were sought for retrieval, and the full texts of 33 articles were assessed for eligibility. Two of these studies were excluded because they did not involve mechanical thrombectomy, one was removed because neither VKAs nor DOACs were used, six were removed because no information was provided regarding antiplatelet or anticoagulant use, three were removed because no information was provided regarding VKA or DOAC use, one was removed because it was not based on original data, three were removed because they did not have comparator arms, one was removed because it used intravenous thrombolysis for comparison, and one was removed because it was a systematic review and meta-analysis. A total of 15 studies remained and were included in the final analysis [5–19]. The study selection flow chart is displayed in Fig. 1.

The characteristics of the enrolled studies are presented in Table 1. All studies were non-randomized and published between 2015 and 2020, with sample sizes ranging from 98 to 6173. The mean ages of the enrolled population ranged from 62 to 79 years, and atrial fibrillation was more common in the VKA and DOAC groups than in the control group. The mean National Institutes of Health Stroke Scale (NIHSS) scores ranged from 10 to 18, with most scores ranging from 14 to 17. Nine studies reported confirmed therapeutic use of OACs, and two of them reported confirmed therapeutic use of DOACs.. Confirmed therapeutic VKA use was defined as an international normalized ratio (INR) of > 1.7, and confirmed therapeutic DOAC use was defined as the intake of one or two doses within the last 24 or 12 h, respectively, or an INR of > 1.2 [20]. All studies reported the outcomes of sICH and functional status at 3 months.

**Table 1:** Characteristics of enrolled studies

Author [Year]	Study Population	Age (mean)	Atrial Fibrillation (N%)	NIHSS (mean)	No. of patients (VKA/DOAC /non-OAC)	Outcome Measure
Seiffge 2015 [5]	patients with AIS occurring while taking NOACs who were treated with IVT or IAT or both	VKA 77 (68–83) DOAC 76 (68–81) Non-OAC 71 (60–79)	VKA 345 (78.8) DOAC 68 (87.2) Non-OAC 2152 (24.3)	VKA 14.5 (7–19) DOAC 14 (8–19) Non-OAC 10 (6–16)	441/78/8838	sICH (ECASS-II), MRS
Rebello 2015 [6]	retrospective analysis of prospectively collected consecutive intra-arterial therapies	VKA 68.7 ± 13.57 DOAC 68.6 ± 7.45 Non-OAC 64.0 ± 14.7	VKA 17 (58) DOAC 14 (82) Non-OAC 66(24)	VKA 19.3 ± 5.5 DOAC 17.2 ± 7.6 Non-OAC 18.3 ± 6.3	29/17/265	sICH (HI-I/PH-II), mortality, reperfusion, MRS
Rozeman 2016 [7]	national Dutch database on IAT in AIS patients	VKA 61.5 (27–80) Non-OAC 62 (12–93)	Not provided	VKA 14.5 (5–38) Non-OAC 16 (1–42)	18/Nil/438	sICH (ECASS-II), MRS
Benavente 2016 [8]	30 APs with current use of dicumarins and 87 N-APs treated with direct MT with stent retriever	VKA 72.8 ± 7.85 Non-OAC 67.07 ± 10.60	VKA 87.49% Non-OAC 17.44%	VKA 17 (7–28) Non-OAC 16 (2–24)	30/Nil/87	sICH (ECASS-II), mortality, reperfusion, MRS
Uphaus 2016 [9]	adult AIS patients who underwent thrombectomy	Not provided	Not provided	Not provided	85/Nil/730	sICH (ECASS-II), MRS
Mundiyanapurath 2016 [10]	patients with anterior circulation stroke treated with endovascular therapy	All 72 (62–79)	Not provided	All 17 (14–20)	45/Nil/390	sICH (ECASS-II), MRS
Zapata-Wainberg 2017 [11]	consecutive patients with AIS treated with MT	OAC 72.73 ± 9.23 Non-OAC 65.87 ± 13.23	OAC 104 (92.0) Non-OAC 96 (24.7)	OAC 16 (9) Non-OAC 17 (9)	104/9/389	sICH (SIST), reperfusion, MRS
Cernik 2018 [12]	consecutive non-selected patients, who were treated with MT	VKA 76 ± 11 DOAC 77 ± 6 Non-OAC 70.0 ± 12.5	OAC 74 (84) Non-OAC 230 (38)	OAC 16.5 (2–36) Non-OAC 17.0 (1–42)	50/15/615	sICH (PH-II, ECASS-II), MRS
L’Allinec 2018 [13]	Aps and N-APs treated with MT, and N-APs treated with IV-rtPA and MT	OAC 75 (13) Non-OAC 64 (14)	OAC 30 (75) Non-OAC 22 (21)	OAC 18 (8) Non-OAC 17 (7)	30/4/105	sICH (NINDS), MRS
Krajickova 2018 [14]	patients with AIS in the anterior circulation due to LVO treated with MT with or without IVT	OAC 75 ± 8.0 Non-OAC 71.1 ± 13.8	OAC 26 (100) Non-OAC 114 (44)	OAC 15 (1–28) Non-OAC 14 (0–40)	21/5/259	sICH (SIST-MOST), MRS
Wong 2018 [15]	consecutive patients undergoing MT	OAC 72.5 (59–78.5) Non-OAC 70.5 (57–76)	OAC 31 (86.1) Non-OAC 28 (42.4)	OAC 16 (9.5–20.5) Non-OAC 16.5 (10–20)	23/13/66	sICH (ECASS-II), reperfusion, MRS
Meinel 2020 [16]	investigate the safety and efficacy of neurothrombectomy device in AIS	VKA 79 (71–84) DOAC 78 (70–83) Non-OAC 73 (60–81)	VKA 176 (80) DOAC 66 (68.0) Non-OAC 641 (40.1)	VKA 16 (11–20) DOAC 16 (8.5–19.5) Non-OAC 16 (10–20)	222/98/1622	sICH (ECASS-II), mortality, MRS
Goldhoorn 2020 [17]	AIS caused by an intracranial anterior circulation occlusion undergoing EVT	OAC 78 (69–84) Non-OAC 71 (60–80)	OAC 394 (78) Non-OAC 359 (13)	OAC 17 (12–20) Non-OAC 16 (11–19)	404/98/2660	sICH (HI-I/PH-II), mortality, reperfusion, MRS
Ramos-Araque 2020 [18]	all consecutive AIS patients treated with reperfusion therapies	VKA 76.66 ± 10.2 DOAC 76.37 ± 9.79 Non-OAC 71.5 ± 13.2	VKA 171 (89) DOAC 78 (96) Non-OAC 153 (13)	VKA 18 (12–21) DOAC 15 (9–20) Non-OAC 16 (10–20)	193/81/1181	sICH (ECASS-II), mortality, MRS
Kupper 2020 [19]	all consecutive patients with LVO with an intention to be treated with EVT	VKA 77.7 ± 10 DOAC 77.7 ± 10.9 Non-OAC 72.0 ± 13.5	VKA 413 (86.9) DOAC 715 (87.5) Non-OAC 1438 (29.9)	VKA 15 (0–42) DOAC 15 (0–42) Non-OAC 14 (0–42)	479/827/4867	sICH (ECASS-II), mortality, reperfusion, MRS

*VKA* Vitamin K antagonist, *DOAC* Direct oral anticoagulant, *OAC* Oral anticoagulant, *NOAC* Novel oral anticoagulant, *IVT* intravenous thrombolysis, *IAT* Intra-arterial thrombolysis, *MT* Mechanical thrombectomy, *EVT* Endovascular thrombectomy, *APs* anticoagulated patients, *AIS* Acute ischemic stroke, *LVO* Large vessel occlusion, *sICH* Symptomatic intracranial hemorrhage, *MRS* Modified Rankin Scale, *ECASS-II* European Cooperative Acute Stroke Study II, *HI-I* Hemorrhagic infarction type 1, *PH-II* Parenchymal hematoma type 2, *SIST-MOST* Safe Implementation of Thrombolysis in Stroke-Monitoring Study, *NINDS* National Institute of Neurological Disorders and Stroke

### Meta-analysis results for safety outcomes

**Fig. 2:**
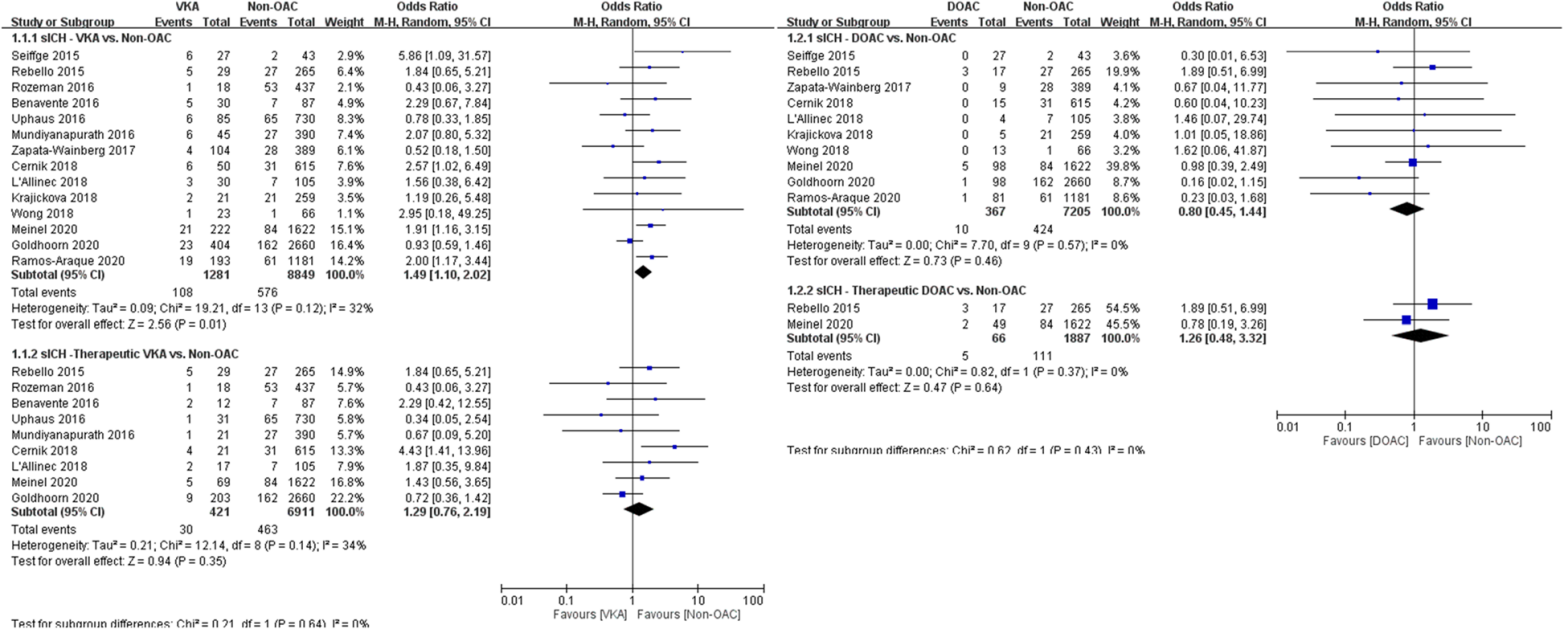
**A** Forest plot for VKA vs. non-OAC regarding sICH. **B** Forest plot for DOAC vs. non-OAC regarding sICH

The primary safety outcome analysis compared 1281 patients who received VKAs and 361 patients who received DOACs with 8849 and 7205 controls, respectively. The VKA group had a higher rate of sICH than the control group (8.4% vs. 6.5%, OR 1.49, 95% CI 1.10–2.02; Fig. 2A). However, this effect was not observed for the DOAC group, in which the sICH risk did not significantly differ from that of the control group (2.7% vs. 5.9%, OR 0.80, 95% CI 0.45–1.44; Fig. 2B). The sICH rates of patients who received a confirmed therapeutic dose of OACs and controls were similar for both VKAs and DOACs (7.1% vs. 6.7%, OR 1.29, 95% CI 0.76–2.19 and 7.6% vs. 5.9%, OR 1.26, 95% CI 0.48–3.32, respectively; Fig. 2A and B).

**Fig. 3:**
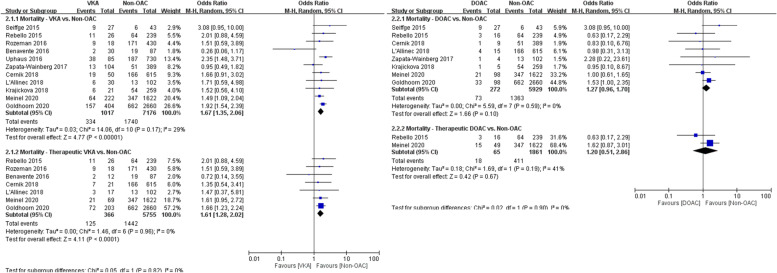
**A** Forest plot for VKA vs. non-OAC regarding mortality. **B** Forest plot for DOAC vs. non-OAC regarding mortality

In the analysis of the secondary safety outcome, 1017 patients who received VKAs and 272 patients who received DOACs were compared with 7176 and 5929 controls, respectively. The mortality rate was higher in the VKA group than in the control group (33% vs. 24%, OR 1.67, 95% CI 1.35–2.06), even in patients who received confirmed therapeutic doses of VKAs (34% vs. 25%, OR 1.61, 95% CI 1.28–2.02; Fig. 3A). Compared with the control group, the mortality rates were similar for patients who received DOACs, regardless of whether they had received confirmed therapeutic doses (28% vs. 22%, OR 1.20, 95% CI 0.51–2.86) or not (27% vs. 23%, OR 1.27, 95% CI 0.96–1.70; Fig. 3B).

### Meta-analysis results for efficacy outcomes

**Fig. 4:**
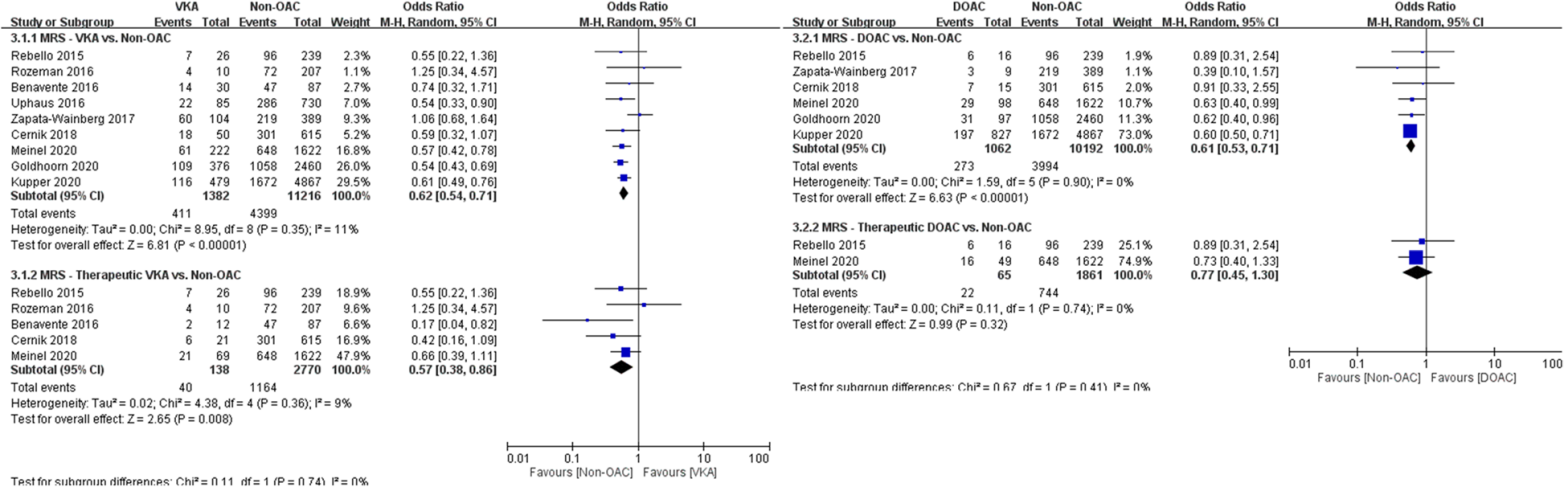
**A** Forest plot for VKA vs. non-OAC regarding functional outcome. **B** Forest plot for DOAC vs. non-OAC regarding functional outcome

In the analysis of the primary efficacy outcome, 1382 patients who received VKAs and 1062 patients who received DOACs were compared with 11,216 and 10,192 controls, respectively. Patients who received OACs had worse functional outcomes (modified Rankin scale score of ≤ 2) at 3 months than controls had, both in the VKA and DOAC groups (30% vs. 39%, OR 0.62, 95% CI 0.54–0.71 and 26% vs. 39%, OR 0.61, 95% CI 0.53–0.71, respectively; Fig. 4A and B). Similar results were observed in comparisons of patients who received confirmed therapeutic doses of VKAs with controls (29% vs. 42%, OR 0.57, 95% CI 0.38–0.86) but not in comparisons of patients who received confirmed therapeutic doses of DOACs with controls, in which no difference in functional outcomes was observed (34% vs. 40%, OR 0.77, 95% CI 0.45–1.30; Fig. 4A and B).

**Fig. 5:**
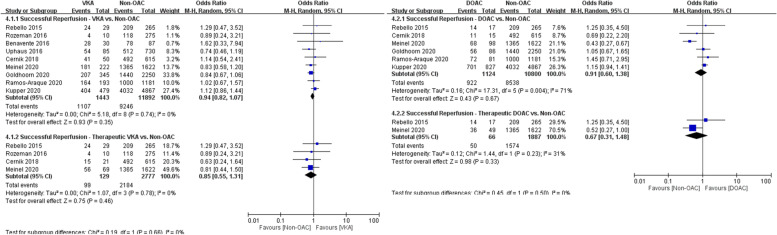
**A** Forest plot for VKA vs. non-OAC regarding successful reperfusion. **B** Forest plot for DOAC vs. non-OAC regarding successful reperfusion

In the analysis of the secondary efficacy outcome, 1443 patients who received VKAs and 1124 patients who received DOACs were compared with 11,892 and 10,800 controls, respectively. Similar rates of successful reperfusion (mTICI score of > 2b) were achieved in the VKA and control groups (76% vs. 78%, OR 0.94, 95% CI 0.82–1.07) and in the DOAC and control groups (82% vs. 79%, OR 0.91, 95% CI 0.60–1.38; Fig. 5A and B). Compared with controls, no differences in successful reperfusion were observed in patients who received confirmed therapeutic doses of VKAs (77% vs. 79%, OR 0.85, 95% CI 0.55–1.31) and DOAC (76% vs. 83%, OR 0.67, 95% CI 0.31–1.48; Fig. 5A and B).

## Discussion

This systematic review and meta-analysis demonstrated that (1) patients with AIS who received EVT and were treated with VKAs had higher risks of sICH and mortality, but such an effect was not observed for patients treated with DOACs. (2) Patients with AIS who underwent EVT and received confirmed therapeutic doses of VKAs had a lower risk of sICH as compared with total VKA patients, but not lower than those without OACs. (3) Positive outcomes were achieved more commonly by patients who did not receive OACs than by those who did. (4) Finally, similar rates of successful reperfusion were achieved by patients who received OACs and those who did not.

sICH is a serious concern in patients receiving EVT and carries the risk of unanticipated complications that can increase mortality. Previous studies have reported varying sICH rates in those receiving EVT, ranging from less than 5% to more than 16% [21, 22]. Studies have reported positive associations of female sex, diabetic mellitus, high blood pressure, lower Alberta Stroke Program Early CT score, and longer treatment interval with the risk of hemorrhage [23–25]. Tirofiban, an antiplatelet agent, was also demonstrated to be associated with an increased risk of sICH in patients receiving EVT [26]. However, this increased risk was not discovered in patients with lower platelet counts [27]. OAC treatment, especially with VKAs, is also controversial because of its potential to increase the risk of hemorrhage.

In this pooled analysis, VKA was associated with a higher risk of sICH. Although some previous research has not observed such an effect, Meinel and Ramos observed an increased risk in studies conducted with large sample sizes [16, 18]. A possible explanation for the higher rate of sICH is that atrial fibrillation, which carries a high risk of hemorrhagic transformation, is more prevalent among patients receiving VKAs. In addition, strong anticoagulant activity may lead to more severe hemorrhages. In most enrolled studies, patients who received therapeutic doses of VKAs did not have a significantly higher risk of sICH. This could be because patients who received therapeutic doses of VKAs may have had lower stroke severity because they received appropriate treatment. However, a similar result was not observed in the DOAC group, which had a similar risk of sICH as patients who did not receive OACs. The steady pharmacokinetic activity of DOACs may explain this result; most patients receive DOACs within the therapeutic range and experience low stroke severity. The lower risk of systemic bleeding of DOACs than of VKAs may also be responsible for this effect [28]. Most patients currently receiving VKAs could gradually shift to DOAC use, except for some patients with contraindications such as antiphospholipid syndrome or receiving mechanical valve transplantation. Increasing DOAC use may diminish the risk of sICH, and operators should be aware of the risk of hemorrhage if patients received VKAs before undergoing EVT procedures.

Mortality after EVT is another safety concern that we assessed. A pooled analysis of 15 studies revealed a mortality rate after EVT of approximately 15% [29]. Factors affecting mortality included age, pretreatment collateral arteries status, baseline blood glucose level, sICH, and baseline NIHSS score [30]. Despite the lack of adjustment for confounding factors in the present meta-analysis, mortality was higher in patients who received VKAs, even among those who received doses within the therapeutic range. The higher mortality rate was correlated with higher sICH risk in patients who received VKAs; this result supports the risk of hemorrhage being related to mortality. Because the rate of sICH was lower in the DOAC group, mortality for patients who received DOACs and those who did not was similar. However, although mortality was higher in patients who received VKAs, the benefits of EVT in AIS with LVO were considerable. Currently, intravenous rtPA is the gold standard therapy for AIS within 4.5 h. Most patients receiving EVT had undergone prior intravenous rtPA treatment, which was once considered a risk factor for intracranial hemorrhage. In our subgroup analysis of patients receiving EVT without concomitant rtPA, the risks of sICH and mortality were higher in the VKA group than the DOAC and control arms. These results were similar to those of the pooled analysis and may indicate that intravenous rtPA is not a major contributor to sICH or mortality risk; however, this hypothesis requires further study.

In a German study of the outcomes of EVT, postprocedural functional independence was achieved by approximately 37% of patients [31]. A similar rate of positive outcomes was achieved in our pooled analysis, but the rate was significantly lower in those who received VKAs. Higher risks of sICH and morality in patients receiving VKAs possibly contributed to this result. Such associations have been reported in several previous studies [32, 33]. Although some studies have suggested that successful reperfusion is also an independent predictor of favorable outcomes, [34] the recanalization rates were similar in our OAC and control arms, indicating that successful reperfusion is not a major contributor.

EVT was introduced nearly 20 years ago, and numerous novel devices have been developed since then; therefore, the successful reperfusion rate has increased over time. In the past, recanalization was thought to be easily achieved in patients with AIS and atrial fibrillation because the blood clots were more friable than those in in patients with atherosclerosis. However, in the present meta-analysis, successful reperfusion was achieved at similar rates in all groups, including patients who received OACs, who were more likely to have atrial fibrillation. Additionally, no differences were observed in the treatment effects of EVT between patients with LVO with or without atrial fibrillation [35]. In our pooled analysis, the recanalization rate was 80%–85%, which is similar to the rates observed in most other studies. However, most studies did not report the number of passes; therefore, it is difficult to draw conclusions regarding successful reperfusion because a higher number of passes increases the risk of vessel damage and hemorrhage.

The heterogeneity of the present meta-analysis was minimal, both in the overall and subgroup analyses, which indicates that our results are reliable. However, the present study has several limitations. First, all enrolled studies were retrospective, indicating that many confounding factors may have required adjustment. However, the present study did not adjust for these confounding factors; thus, the results should be interpreted with caution. Second, heterogeneity across studies is inevitable. Differences in populations, underlying diseases, device usage, and outcome measurement tools could influence the results, but such influence was minimized by statistical methods with the use of the random-effects model. Third, atrial fibrillation, which carries a high risk of hemorrhage, was more common in patients who received OACs than in those who did not, and this may have influenced the results. Those who received OACs may also have had comorbidities such as autoimmune disorders. This effect could not be adequately adjusted for in most enrolled studies. Finally, the dosage of OAC was not frequently reported. A low therapeutic dose is correlated with poor disease control, which may lead to a high risk of hemorrhage and mortality. Although the present study conducted a subgroup analysis for patients with and without confirmed therapeutic doses of OACs, the available data were still inadequate. Besides, the use of idarucizumab for dabigatran reversal was also not reported in enrolled studies, which was a relevant contributor to the risk of hemorrhage. However, despite these limitations, a higher risk of sICH was observed in patients with AIS receiving EVT who used VKAs compared with those who used DOACs or did not receive OACs. Further research is warranted.

## Conclusions

In patients with AIS who received OACs treatment and EVT, VKA use was associated with a higher risk of sICH and mortality comparing with DOAC. In those treated with therapeutic doses of OACs, the risks of sICH and mortality were lower. Patients who did not receive OACs exhibited more favorable outcomes than those who did; this result was possibly related to the risk of sICH. The successful reperfusion rate did not differ between groups. Although anticoagulant use was associated with worse outcome, these data did not examine the treatment effect of EVT versus medical therapy.

## Supplementary Information

**Additional file 1:** **Supplementary Table 1. **Search strategy.** Supplementary Figure 1. **Risk of bias assessment. **Supplementary Figure 2. **Funnel plot for VKA/DOAC vs. non-OAC regarding sICH. **Supplementary Figure 3. **Funnel plot for VKA/DOAC vs. non-OAC regarding mortality. **Supplementary Figure 4. **Funnel plot for VKA/DOAC vs. non-OAC regarding functional outcome. **Supplementary Figure 5. **Funnel plot for VKA/DOAC vs. non-OAC regarding successful reperfusion.  

## Data Availability

Data and statistical analysis are available upon request to corresponding author.

## Abbreviations

AIS: Acute ischemic stroke
CIs: Confidence intervals
DOACs: Direct oral anticoagulants
EVT: Endovascular thrombectomy
INR: International normalized ratio
LVO: Large vessel occlusion
mTICI: Modified treatment in cerebral infarction
NIHSS: National Institutes of Health Stroke Scale
OACs: Oral anticoagulants
rtPA: Recombinant tissue plasminogen activator
sICH: Symptomatic intracranial hemorrhage
VKAs: Vitamin K antagonists
