# Supplementary material for: Safety and efficacy of endovascular thrombectomy in acute ischemic stroke treated with anticoagulants: a systematic review and meta-analysis
Source: Thromb J. 2022 Jun 21;20:35. doi: 10.1186/s12959-022-00394-y (PMC9210791; doi:10.1186/s12959-022-00394-y)

***Supplementary Table 1. Search strategy***

**Pubmed**

| **P**  Population / Patient (problem, disease, coexisting problems) | ("endovascular procedures"[MeSH Major Topic] OR endovascular[Title/Abstract] OR endovascular procedure[Title/Abstract] OR endovascular treatment[Title/Abstract] OR intervention[Title/Abstract] OR interventional neuroradiology[Title/Abstract] OR intra-arterial[Title/Abstract] OR intra-arterial treatment[Title/Abstract] OR thrombolytic[Title/Abstract] OR thrombolytic therapy[Title/Abstract] OR thrombectomy[Title/Abstract] OR mechanical thrombectomy[Title/Abstract] OR aspiration[Title/Abstract] OR stent retriever[Title/Abstract] OR large vessel[Title/Abstract] OR large vessel occlusion[Title/Abstract] OR reperfusion[Title/Abstract] OR recanalization[Title/Abstract] OR revascularization[Title/Abstract]) |
| --- | --- |
| **I**  Intervention I (Therapy) | (anticoagulants[MeSH Terms] OR anticoagulants OR anticoagulation[Title/Abstract] OR anticoagulation therapy[Title/Abstract] OR direct oral anticoagulants[Title/Abstract] OR DOAC[Title/Abstract] OR novel oral anticoagulants[Title/Abstract] OR NOAC[Title/Abstract] OR non-vitamin K antagonist oral anticoagulants[Title/Abstract] OR vitamin K antagonists[Title/Abstract] OR VKA[Title/Abstract] OR coumarin[Title/Abstract] OR warfarin[Title/Abstract] OR internationalized normalized ratio[Title/Abstract] OR INR[Title/Abstract]) |
| **C**  Comparison | / |
| **O**  Outcome | (intracerebral hemorrhage[Title/Abstract] OR intracerebral haemorrhage[Title/Abstract] OR intracranial hemorrhage[Title/Abstract] OR intracranial haemorrhage[Title/Abstract] OR symptomatic intracerebral hemorrhage[Title/Abstract] OR symptomatic intracerebral haemorrhage[Title/Abstract] OR symptomatic intracranial hemorrhage[Title/Abstract] OR symptomatic intracranial haemorrhage[Title/Abstract] OR hematoma[Title/Abstract]) |

**Embase**

| **P**  Population / Patient (problem, disease, coexisting problems) | ‘endovascular’:ab,ti OR ‘endovascular procedure’:ab,ti OR ‘endovascular treatment’:ab,ti OR ‘intervention’:ab,ti OR ‘interventional neuroradiology’:ab,ti OR ‘intra-arterial’:ab,ti OR ‘intra-arterial treatment’:ab,ti OR ‘thrombolytic’:ab,ti OR ‘thrombolytic therapy’:ab,ti OR ‘thrombectomy’:ab,ti OR ‘mechanical thrombectomy’:ab,ti OR ‘aspiration’:ab,ti OR ‘stent retriever’:ab,ti OR ‘large vessel’:ab,ti OR ‘large vessel occlusion’:ab,ti OR ‘reperfusion’:ab,ti OR ‘recanalization’:ab,ti OR ‘revascularization’:ab,ti |
| --- | --- |
| **I**  Intervention I (Therapy) | ‘anticoagulants’:ab,ti OR ‘anticoagulation’:ab,ti OR ‘anticoagulation therapy’:ab,ti OR ‘direct oral anticoagulants’:ab,ti OR ‘DOAC’:ab,ti OR ‘novel oral anticoagulants’:ab,ti OR ‘NOAC’:ab,ti OR ‘non-vitamin K antagonist oral anticoagulants’:ab,ti OR ‘vitamin K antagonists’:ab,ti OR ‘VKA’:ab,ti OR ‘coumarin’:ab,ti OR ‘warfarin’:ab,ti OR ‘internationalized normalized ratio’:ab,ti OR ‘INR’:ab,ti |
| **C**  Comparison | / |
| **O**  Outcome | ‘intracerebral hemorrhage’:ab,ti OR ‘intracerebral haemorrhage’:ab,ti OR ‘intracranial hemorrhage’:ab,ti OR ‘intracranial haemorrhage’:ab,ti OR ‘symptomatic intracerebral hemorrhage’:ab,ti OR ‘symptomatic intracerebral haemorrhage’:ab,ti OR ‘symptomatic intracranial hemorrhage’:ab,ti OR ‘symptomatic intracranial haemorrhage’:ab,ti OR ‘hematoma’:ab,ti |

**Cochrane**

| **P**  Population / Patient (problem, disease, coexisting problems) | endovascular procedures OR endovascular OR endovascular procedure OR endovascular treatment OR intervention OR interventional neuroradiology OR intra-arterial OR intra-arterial treatment OR thrombolytic OR thrombolytic therapy OR thrombectomy OR mechanical thrombectomy OR aspiration OR stent retriever OR large vessel OR large vessel occlusion OR reperfusion OR recanalization OR revascularization |
| --- | --- |
| **I**  Intervention I (Therapy) | anticoagulants OR anticoagulants OR anticoagulation OR anticoagulation therapy OR direct oral anticoagulants OR DOAC OR novel oral anticoagulants OR NOAC OR non-vitamin K antagonist oral anticoagulants OR vitamin K antagonists OR VKA OR coumarin OR warfarin OR internationalized normalized ratio OR INR |
| **C**  Comparison | / |
| **O**  Outcome | intracerebral hemorrhage OR intracerebral haemorrhage OR intracranial hemorrhage OR intracranial haemorrhage OR symptomatic intracerebral hemorrhage OR symptomatic intracerebral haemorrhage OR symptomatic intracranial hemorrhage OR symptomatic intracranial haemorrhage OR hematoma |

***Supplementary Figure 1. Risk of bias assessment***


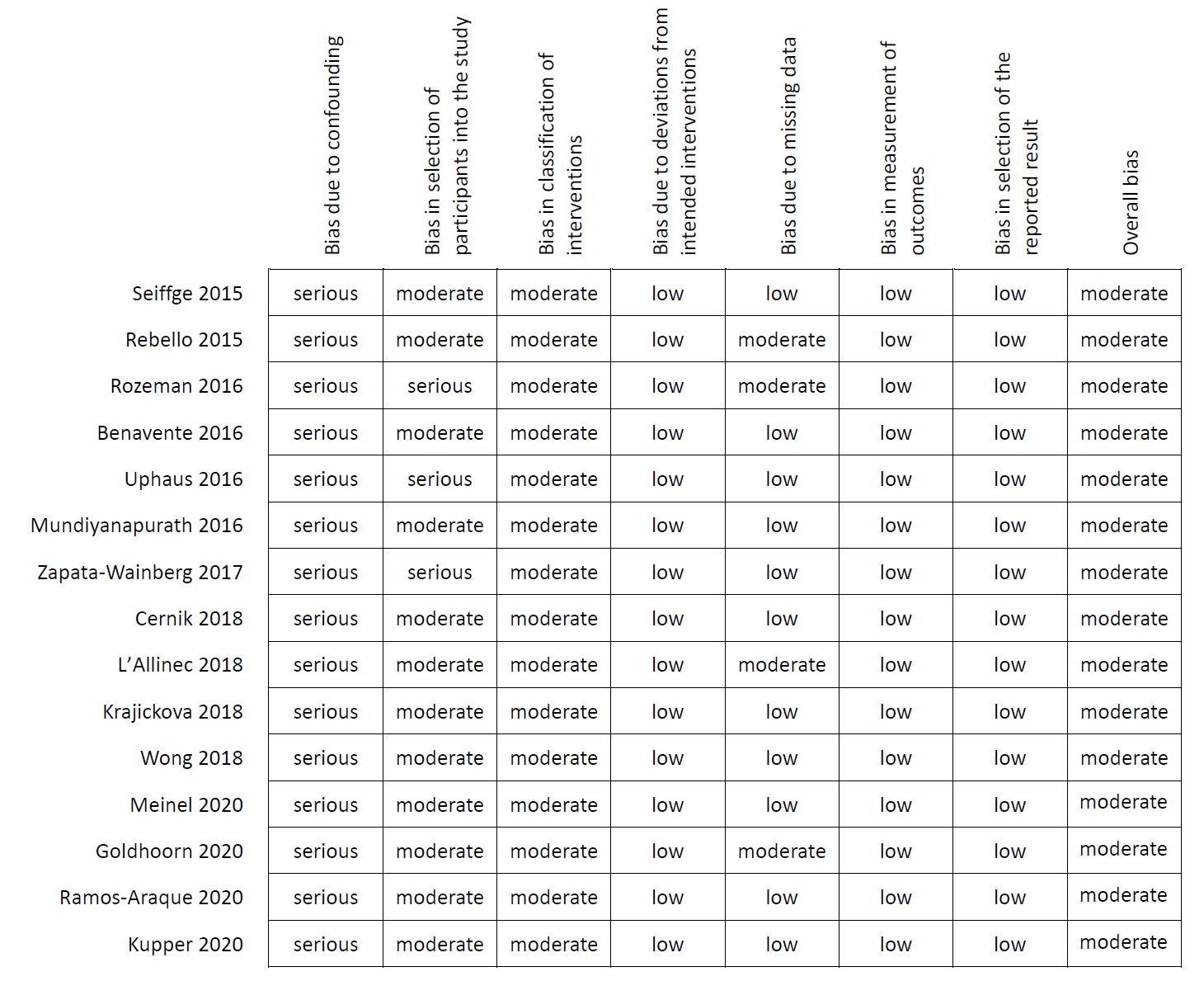


***Supplementary Figure 2. Funnel plot for VKA/DOAC vs. non-OAC regarding sICH***


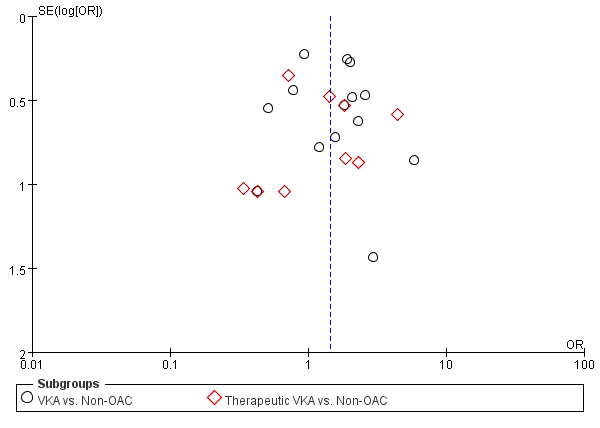

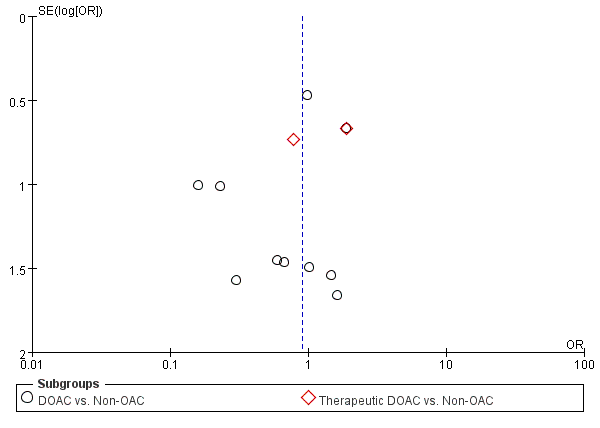


***Supplementary Figure 3. Funnel plot for VKA/DOAC vs. non-OAC regarding mortality***


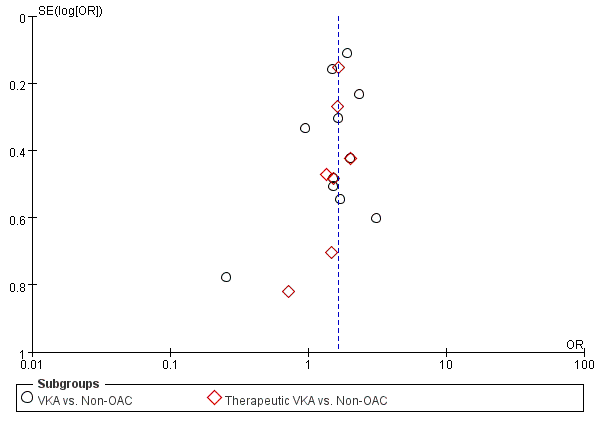

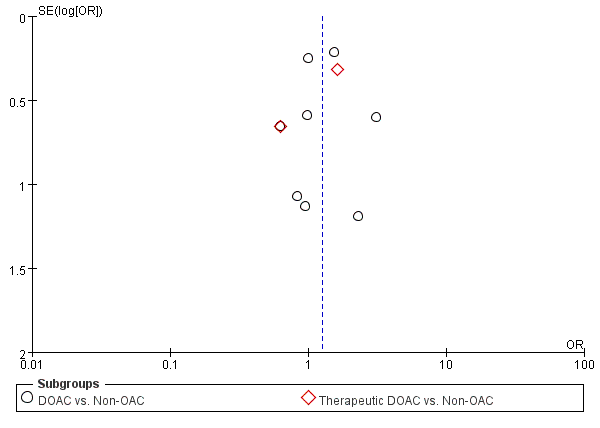


***Supplementary Figure 4. Funnel plot for VKA/DOAC vs. non-OAC regarding functional outcome***


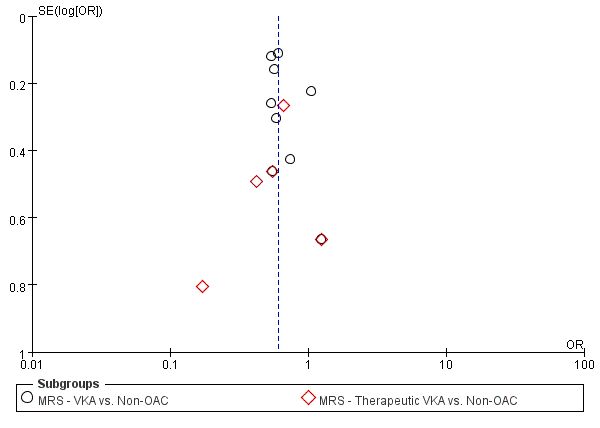

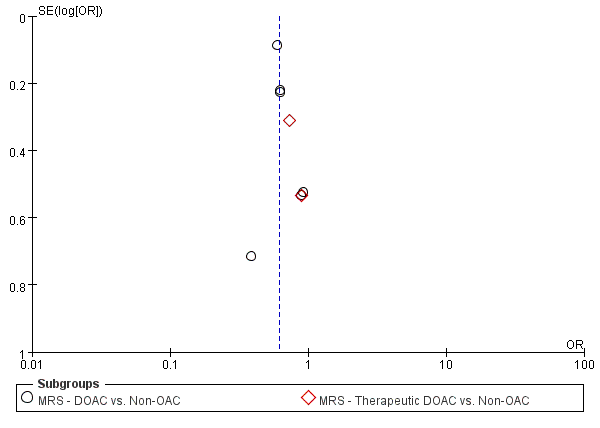


***Supplementary Figure 5. Funnel plot for VKA/DOAC vs. non-OAC regarding successful reperfusion***


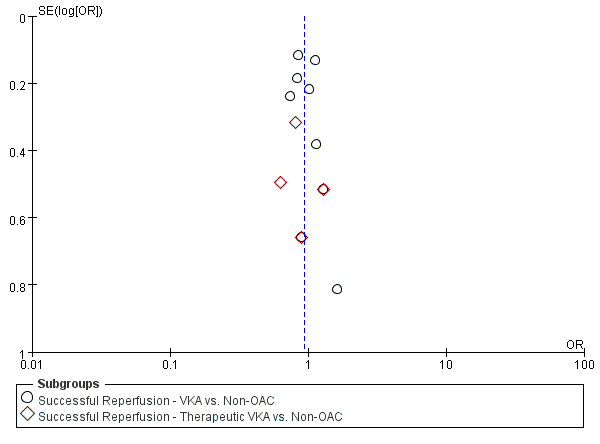

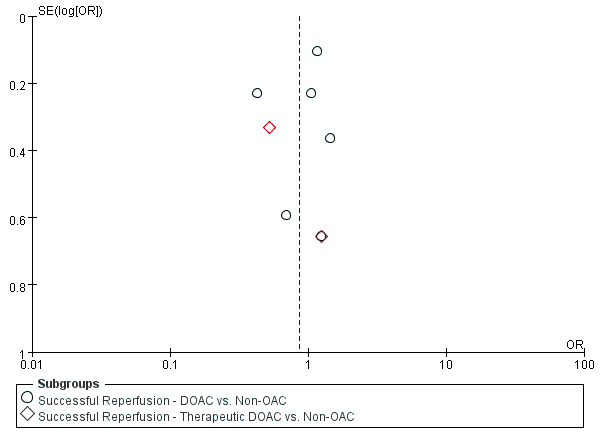

Supplement: Supplementary file 1 — Additional file 1: Supplementary Table 1. Search strategy. Supplementary Figure 1. Risk of bias assessment. Supplementary Figure 2. Funnel plot for VKA/DOAC vs. non-OAC regarding sICH. Supplementary Figure 3. Funnel plot for VKA/DOAC vs. non-OAC regarding mortality. Supplementary Figure 4. Funnel plot for VKA/DOAC vs. non-OAC regarding functional outcome. Supplementary Figure 5. Funnel plot for VKA/DOAC vs. non-OAC regarding successful reperfusion. [file 12959_2022_394_MOESM1_ESM.docx]
